# Performance and Agreement Between WGS Variant Calling Pipelines Used for Bovine Tuberculosis Control: Toward International Standardization

**DOI:** 10.3389/fvets.2021.780018

**Published:** 2021-12-14

**Authors:** Víctor Lorente-Leal, Damien Farrell, Beatriz Romero, Julio Álvarez, Lucía de Juan, Stephen V. Gordon

**Affiliations:** ^1^VISAVET Health Surveillance Center, Universidad Complutense de Madrid, Madrid, Spain; ^2^Animal Health Department, Faculty of Veterinary Medicine, Universidad Complutense de Madrid, Madrid, Spain; ^3^UCD School of Veterinary Medicine, University College Dublin, Dublin, Ireland

**Keywords:** whole genome sequencing (WGS), bioinformatics, variant calling pipeline, SNP analysis, genomic epidemiology, Bovine Tuberculosis (bTB), *Mycobacterium bovis*, *Mycobacterium tuberculosis* complex (MTBC)

## Abstract

Whole genome sequencing (WGS) and allied variant calling pipelines are a valuable tool for the control and eradication of infectious diseases, since they allow the assessment of the genetic relatedness of strains of animal pathogens. In the context of the control of tuberculosis (TB) in livestock, mainly caused by *Mycobacterium bovis*, these tools offer a high-resolution alternative to traditional molecular methods in the study of herd breakdown events. However, despite the increased use and efforts in the standardization of WGS methods in human tuberculosis around the world, the application of these WGS-enabled approaches to control TB in livestock is still in early development. Our study pursued an initial evaluation of the performance and agreement of four publicly available pipelines for the analysis of *M. bovis* WGS data (vSNP, SNiPgenie, BovTB, and MTBseq) on a set of simulated Illumina reads generated from a real-world setting with high TB prevalence in cattle and wildlife in the Republic of Ireland. The overall performance of the evaluated pipelines was high, with recall and precision rates above 99% once repeat-rich and problematic regions were removed from the analyses. In addition, when the same filters were applied, distances between inferred phylogenetic trees were similar and pairwise comparison revealed that most of the differences were due to the positioning of polytomies. Hence, under the studied conditions, all pipelines offer similar performance for variant calling to underpin real-world studies of *M. bovis* transmission dynamics.

## Introduction

Animal tuberculosis (aTB) is a chronic infectious disease that affects a wide variety of mammalian species, which is caused by members of the *Mycobacterium tuberculosis* complex (MTBC) (1). The principal agent of TB in cattle (bovine TB, bTB) is *Mycobacterium bovis*. In this manuscript, we will use aTB to refer to TB across wild and domestic animals, and bTB to refer specifically to TB in cattle.

Bovine TB is subjected to control and eradication programmes in many countries, not only due to its economic impact, as a result of reduced yields and animal mortality, but also because of the risk of zoonotic transfer of infection from affected animals to humans (2). Eradication programmes are usually based on a test and slaughter strategy in which cattle that are positive to an official immunological test, such as the intradermal tuberculin test, are culled (3–5). In order to confirm the presence of MTBC species, tissues from the affected animals are cultured in the laboratory (6). In order to eradicate bTB, breakdown events not only need to be detected but also studied for epidemiological links, a process that is greatly facilitated by the application of molecular genetic methods. Due to the clonal structure and limited genetic variability of MTBC species, based on the observed genetic differences between the strains isolated from the breakdown herd and from other aTB episodes, authorities can establish if the outbreak originated from cattle movement, residual infection or contact with wild animal reservoirs (7).

Traditionally, molecular epidemiological studies of aTB are based on techniques that analyse small fragments of the microbial genome, such as spoligotyping or mycobacterial interspersed repeat unit-variable number of tandem repeats (MIRU-VNTR) (8, 9). Although useful in large-scale studies (10–12), some of these methods are laborious and the use of a limited number of loci entails a higher risk of homoplasies and a lack of resolution, limiting their use in the study of local transmission events (13, 14).

The advent of Whole Genome Sequencing (WGS) has revolutionized the study of microbial populations. When applied to epidemiological studies, the availability of the whole genome of the microorganism of interest allows for a much higher resolution than that obtained with previous molecular techniques (15). As a result, the use of WGS in human TB outbreak investigations has rapidly increased in the last decade (16–18).

Due to the limited genetic diversity in MTBC genomes, the standard workflow in MTBC studies is based on the alignment of genomic sequences to a reference genome followed by the detection of genomic variants, usually single nucleotide polymorphisms (SNPs) (19). The procedure starts with genomic DNA extraction, usually through phenol-chloroform or CTAB extraction, library preparation and sequencing using short read sequencing technologies, followed by short-read mapping to the reference genome and variant calling. Variants are then filtered according to certain thresholds and parameters such as proximity to other SNPs, mapping quality, base depth or strand bias. Remaining SNPs are generally concatenated into multi-FASTA files representing multiple sequence alignments and a phylogeny is reconstructed based on SNP differences.

There are several variant calling pipelines for human tuberculosis and, recently, several efforts have been made to assess their performance in human TB outbreak investigations (19–21). Regarding the veterinary field, there is a growing interest in the use of WGS for the analysis of bTB breakdowns, which has resulted in an increasing number of studies being published around the globe (22–26). Nevertheless, although several variant calling pipelines have been developed or are in the making, there are no tool-specific publications and there is a lack of information regarding their overall performance. The aim of this study was to evaluate similarities in design and performance of publicly available variant calling pipelines currently used in laboratories tasked with the application of WGS technologies for aTB eradication.

## Materials and Methods

### Artificial Genome and Read Generation

**Figure 1:**
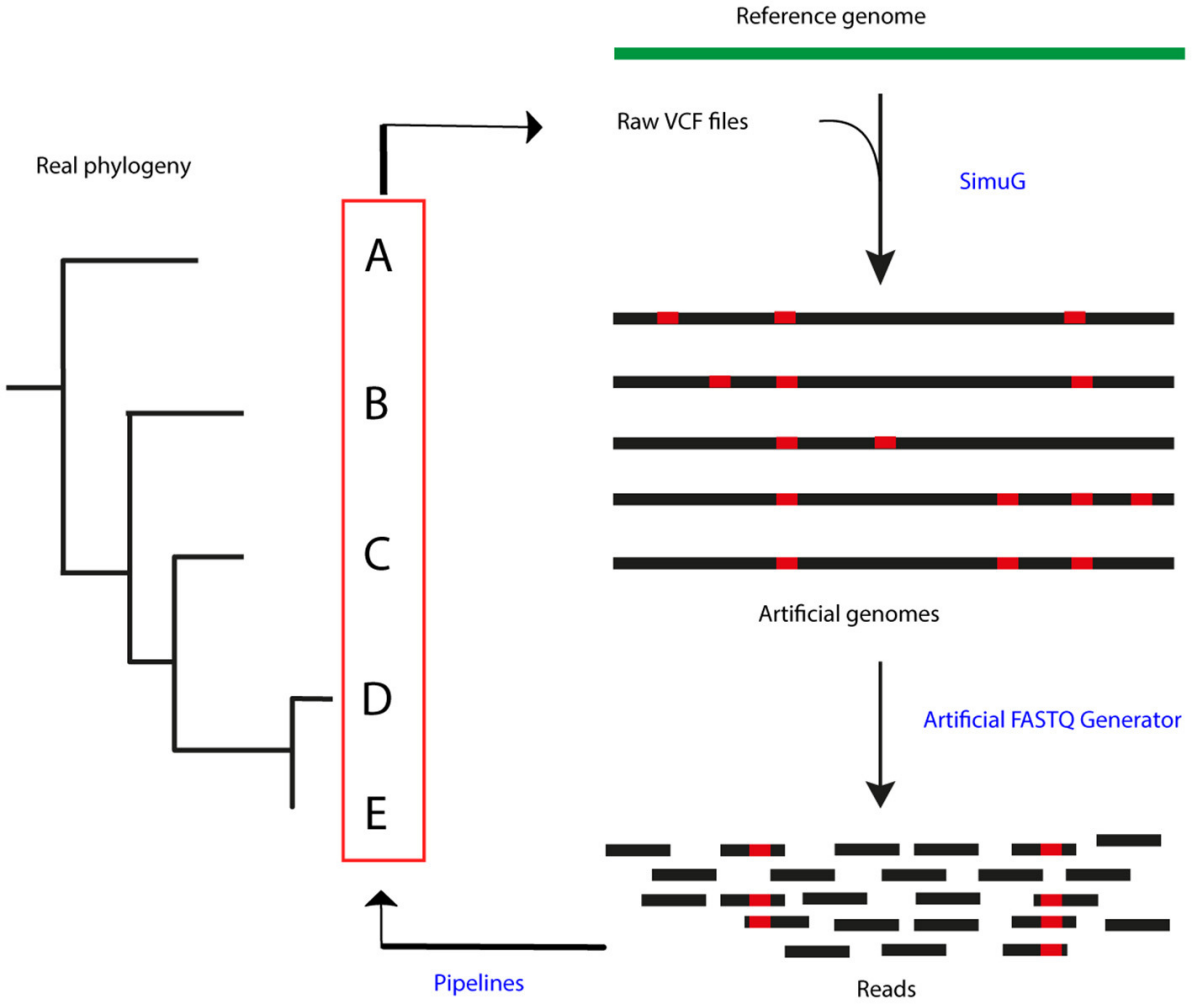
Process summary. Raw VCF files obtained from a real-life phylogeny (22) were used to generate artificial genomes using SimuG and the *M. bovis* AF2122/97 reference genome. Artificial FASTQ Generator was then used to generate artificial reads and these were then input in the evaluated pipelines. Identified variants and output phylogenetic trees were compared between pipelines and the simulation.

In order to simulate a reference phylogeny, raw Variant Call Format (VCF) files were selected from an already published dataset from a bTB high prevalence setting in the Republic of Ireland (22). A total of 47 samples, including two outgroup isolates (isolates 161 and 182), were used to generate artificial mutant genomes by transferring the identified SNPs in the raw VCF files to the *M. bovis* AF2122/97 genome (NCBI RefSeq accession number: NC_002945.4) using simuG 1.0.0 (27) (Figure 1). ArtificialFASTQGenerator 1.0 was then used to generate artificial paired-end reads from the simulated genomes. Several parameters were tested to guarantee a full genome coverage and varying read depth across the whole sequence. Read length was set to 250 bp, template length mean to 650 bp (S.D. = 60), and peak coverage mean for a region was set to 250 (Standard Deviation or S.D. = 0.2) (28). Read qualities were obtained from real-life FASTQ files originating from other sequencing projects (unpublished) and sequencing errors were simulated based on these quality profiles.

### Variant Calling

The artificially generated reads were analyzed with four variant calling pipelines: vSNP 2.03 (25), SNiPgenie 0.5.0, BovTB 20.4, and MTBseq 1.03 (29). The three first pipelines are used for analysis of aTB isolates in the USA (vSNP), Ireland (SNiPGenie), and UK (BovTB); MTBSeq was added as a comparator from the human TB field. Information regarding these pipelines is summarized in Table 1 and described in Supplementary Materials and methods. All pipelines, with exception of SNiPgenie, were run using default settings in miniconda 4.9.2 in Ubuntu 18.04 and in Brigit, the HPC server of the Computer Services at Universidad Complutense of Madrid, using the default reference sequence of *M. bovis* AF2122/97 (NC_002945.4 or LT708304). In SNiPgenie, minimum mapping quality was set to 60 in variant calling and the minimum MQ was set to 30 in posterior filtering steps. Amended variant tables returned by MTBseq were converted to the VCF format using an in-house script for further comparisons. VCF files were generated from excel tables output by vSNP's step 2 script using an in-house script and including a zero coverage VCF of *M. bovis* AF2122/97 in order to include non-parsimonious SNPs.

**Table 1:** Pipeline properties of the different tools evaluated in this study.

	**Pipeline**			
	**vSNP**	**SNiPgenie**	**BovTB**	**MTBseq**
Institution	USDA-APHIS	UCD	APHA	LLI – RCB
Language	Python	Python	Nextflow	Perl
Reference	NC_002945.4	LT708304.1	LT708304.1	NC_002945.4
Parameter setup	No^a^	Yes	No	Yes
**Pre-process**				
Deduplication	Picard	No	FastUniq	Picard
Trimming	None	Yes^b^	Trimmomatic	None
**Mapping and SNP calling**				
Read aligner	BWA	BWA	BWA	BWA
SNP calling	FreeBayes	BCFtools	BCFtools	SAMtools + GATK
Phred base quality	20 (Step 1)	User defined	10	20
Normalize	No	No	Yes	Yes
SNP quality threshold	150	≥40 or User defined	None	None
Min. map quality	56	60	None	None
SNP coverage depth	None	30	5	4F and 4R
Region filter	Excel file (validated problematic positions)	BED file (PE/PPE genes)	TSV (95% similarity self-BLAST)	TSV file (repetitive sequences)
Proximality filter	None	Yes	None	Yes
Allele frequency/fraction	0.05	DP4>4	≥ 0.8	75%
Considers as diploid	Yes	No	No	No
Low coverage positions	Reference if QUAL <50 N if 50 < QUAL <150	Reference	Reference	Consensus base or ignore position if quality is below thresholds in >5% of samples
Alignment file	Core SNPs (polymorphic)	Core SNPs (polymorphic)	Consensus genome	Core SNPs (all)
Spoligotyping	Yes	Yes	No	No
Tree building	RAxML	RAxML	No	No
GUI	No	Yes	No	No
Other analyses	Lineage classification	INDEL analysis	Lineage classification	Lineage classification, antibiotic resistance annotation

a *Only allows for minor parameter settings, such as reference file or type of analysis in step 2*.

b *Deactivated by default*.

### Pipeline Performance Evaluation

As well as a FASTA file containing the artificial genome, SimuG generates a VCF file that contains all the variants included in the generated genome. These artificial VCF files were used as a reference standard to compare the VCF files output from the variant calling pipelines using the Haplotype Comparison Tools 0.3.12 (Som.py).

Variants occurring in locations where no mutations existed in the simulated genome were considered “false positive SNPs,” while mutations not detected by a given pipeline were considered “false negative SNPs”.

The evaluated performance parameters were relative sensitivity or recall rate (true positives/true positives + false negatives) and relative specificity or precision (true positives/true positives + false positives). In addition, alternative (ALT) alleles were extracted from all sample VCF files obtained from each pipeline and combined to obtain the total amount of alleles identified per pipeline. The agreement between the different pipelines was then evaluated using Venn diagrams generated using VennDiagram v1.6.20 in R 3.6.3 (30).

In order to identify groups of genetic elements that usually give rise to false positive and negative calls, all VCF files were annotated using SnpEff 4.3t (31). These genetic elements were then divided into three categories: PE and PPE gene families, mobile genetic elements and other elements (including Direct Repeats and the *pks*12 gene), and their positions in the reference genome were extracted from the GFF3 annotation file available at the NCBI.

Performance was re-evaluated using different levels of hard filtering: (A) unfiltered, (B) a proximal window distance of 10 bp (22), (C) 10 bp window and pipeline default filters, (D) 10 bp window and PE/PPE family proteins, (E) 10 bp window, PE/PPE family proteins and mobile genetic elements, (F) 10 bp window, PE/PPE family proteins, mobile elements and others, and (G) PE/PPE family proteins, mobile elements and others. In order to assess the agreement between pipelines and the accuracy of these results with respect to the original simulated files, filtered positions were also removed from the simulated VCF files.

In addition, the effect of filtering on the number of identified homoplasies was assessed using HomoplasyFinder (32).

### Evaluation of Phylogenetic Outputs and Epidemiological Conclusions

All pipelines, except for BovTB, generate a multi-FASTA alignment containing the concatenated variants. The SNPs in the alignment files obtained from vSNP and SNiPgenie only include polymorphic sites, whereas MTBseq alignments also include monomorphic sites. BovTB yields a consensus genome generated from the VCF files using the BCFtools consensus caller. In order to compare the different methods, core polymorphic SNPs were extracted from these consensus genomes using SNP-sites 2.5.1 (33). In addition, concatenated multi-FASTA files containing polymorphic SNPs were generated for the simulated VCFs using an in-house script.

Maximum-likelihood trees were reconstructed from the resulting multi-FASTA alignment files using RAxML 8.2.12 with 100 bootstraps and the GTRCATI model (34). The bipartitions and best trees obtained from each pipeline were evaluated using Robinson-Foulds (RF) distances and Ward's method for clustering through Treespace in R; briefly, RF pairwise distances between trees were decomposed into a low-dimensional space using a principal coordinate analysis (35). Trees obtained from hard filters that produced the best results in the performance evaluation were compared in a pairwise manner with the simulated phylogeny using Phytools 0.7.82 in R (36).

## Results

### Artificial Read and Genome Simulation

An average of 2.5 x 10^6^ reads (coefficient of variation or C.V. = 0.09%) were generated with an average depth of coverage of 145, with a minimum of 0 and a maximum of 310 reads per site.

Excluding the outgroup isolates, the average observed differences between isolates in the unfiltered simulation was 38.61, with an inter quartile range of 30–47, and a minimum and maximum number of 12 and 64 nucleotide differences, respectively.

### Pipeline Performance

**Figure 2:**
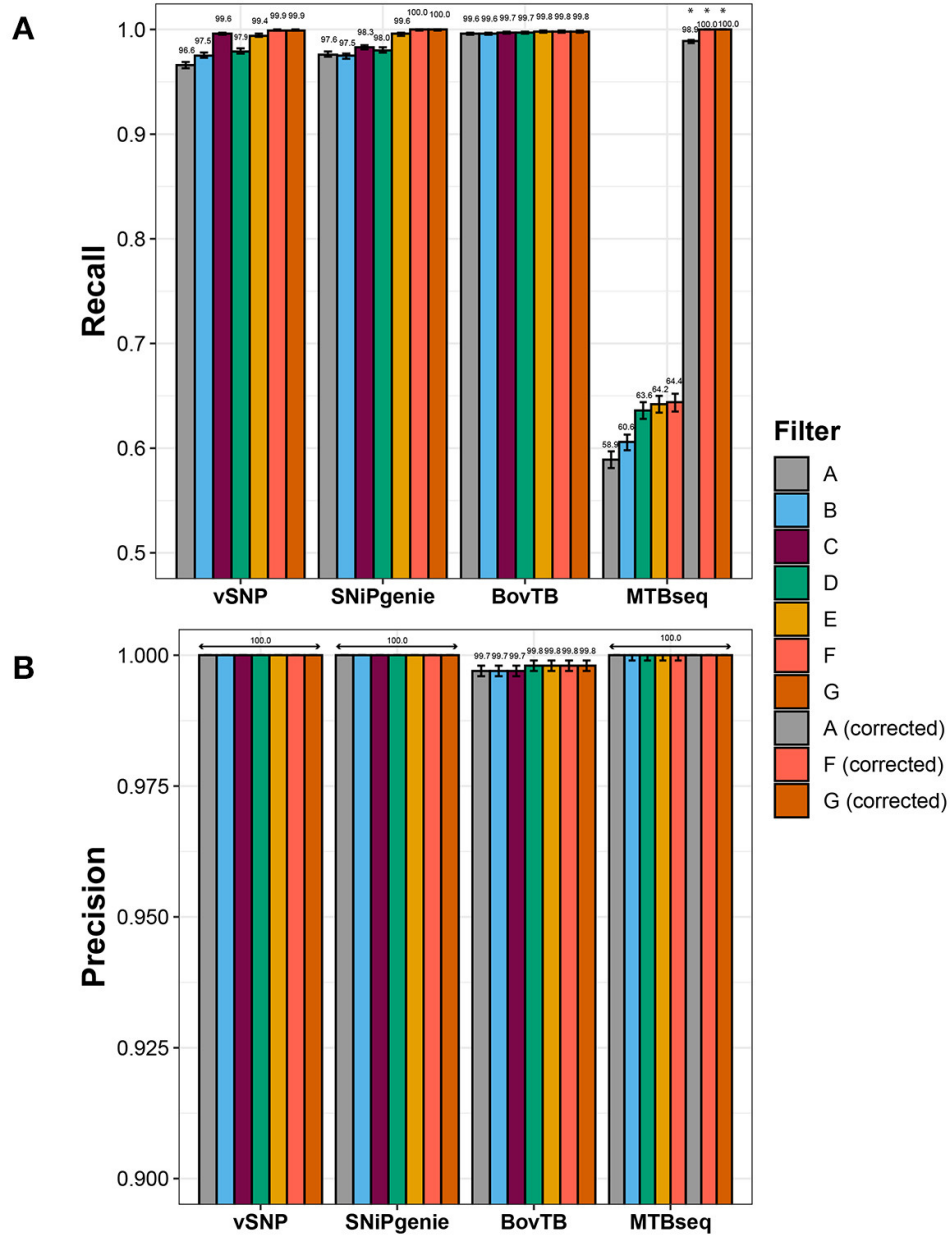
Effect of hard filtering in the performance of the evaluated pipelines when compared to the simulated dataset as indicated in the **(A)** recall and **(B)** precision rates. Asterisks indicate filters in MTBseq for which the minimum coverage threshold was adjusted.

Recall rates were highest for SNiPgenie and BovTB when base parameters were employed, followed by vSNP and MTBseq (Figure 2A).

Sensitivity increased when increasing levels of hard filtering were applied for vSNP, SNiPgenie, and MTBseq, and remained similar for BovTB (Figure 2A). The positive effect was higher when pipeline-specific hard filters were used, in comparison to a proximal window alone (filter B) and a proximal window with additional PE/PPE filtering (filter D). However, recall rates of default filters (filter C) were slightly lower in comparison to the removal of combined proximal SNPs, loci encoding PE/PPE family proteins, mobile elements and other repetitive sequences (filter F) or PE/PPE family proteins, mobile elements and other repetitive sequences (filter G). This was specially the case for SNiPgenie, for which sensitivity increased to levels similar to vSNP and BovTB when filters E, F, or G were applied.

Sensitivity remained below 65% for MTBseq despite the removal of problematic regions. Evaluation of the alignment files for this pipeline revealed that the increased amount of False Negative (FN) calls was produced by strand bias introduced by the artificial read generation, leading to forward or reverse read coverage being below the default minimum threshold (*n* = 4). Adjusting this threshold increased recall rates above 99% (Figure 2A).

After correction of MTBseq parameters, erroneous calls were further evaluated among the unfiltered pipeline results. FN calls were distributed unevenly among the simulated sequences (Supplementary Figure 1) and were mostly located within or near repetitive sequences (data not shown). More than half of the FN positions were shared by at least two of the pipelines, whereas 23 and 20% of the FN positions were identified only by BovTB and vSNP, respectively (Supplementary Figure 2). In addition, the majority of FN positions identified by BovTB in one sample were correctly detected as true SNPs in a varying number of samples (Supplementary Figure 1C).

A small proportion of false positive (FP) SNPs were identified by BovTB (43 SNPs across 37 positions) but, nevertheless, precision was high (>99%) for all of the evaluated pipelines (Figure 2B). Approximately 40% of FPs were located in repetitive regions and, although filtering improved precision in these cases, false positive SNPs were still detected (data not shown). Further analysis of the VCF files in BovTB revealed that the affected positions presented mixed calls caused by artificial sequencing errors. These positions were identified as both FNs and FPs by the Haplotype Caller and were appropriately removed by BovTB in later stages of the analysis. As a result, these mixed positions were ignored in the rest of the comparisons.

**Figure 3:**
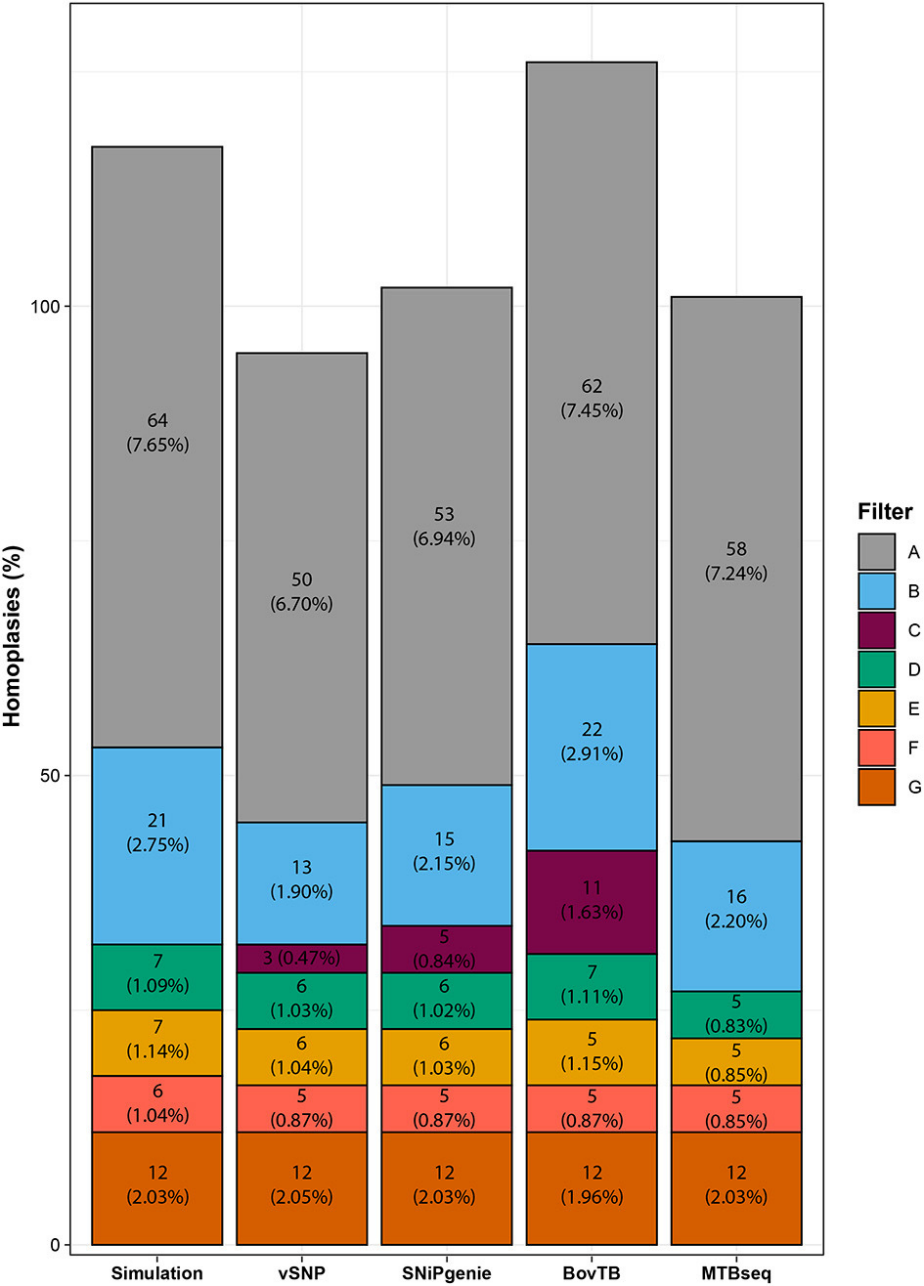
Effect of filtering in the total number of homoplasic positions per pipeline identified by HomoplasyFinder. The percentage represents the proportion of homoplasic positions from the total number of identified positions using each specific filter.

HomoplasyFinder identified 64 (7.65%) homoplasic positions among the generated sequences (Figure 3), mostly located within PE/PPE family proteins, intragenic regions or the *pks12* gene (data not shown). A similar proportion (6.70–7.45%) of homoplasies was identified in the alignments obtained from all of the evaluated pipelines. The removal of proximal SNPs reduced homoplasies to an average of 2%, similarly to what was observed for the removal of all of the problematic regions (filter G). Filtering of problematic regions with the proximity filter produced an additional reduction to 1%; most of the reduction was obtained with the removal of PE/PPE proteins alone and additional filters did not decrease the proportion significantly. Once all filters had been applied, all pipelines presented a reduced proportion of homoplasies compared to the ones present in the simulation. Finally, the use of default filters had a varying effect in the proportion of homoplasies, with vSNP and SNiPgenie obtaining the highest reduction in homoplasic positions.

### Pipeline Agreement

**Figure 4:**
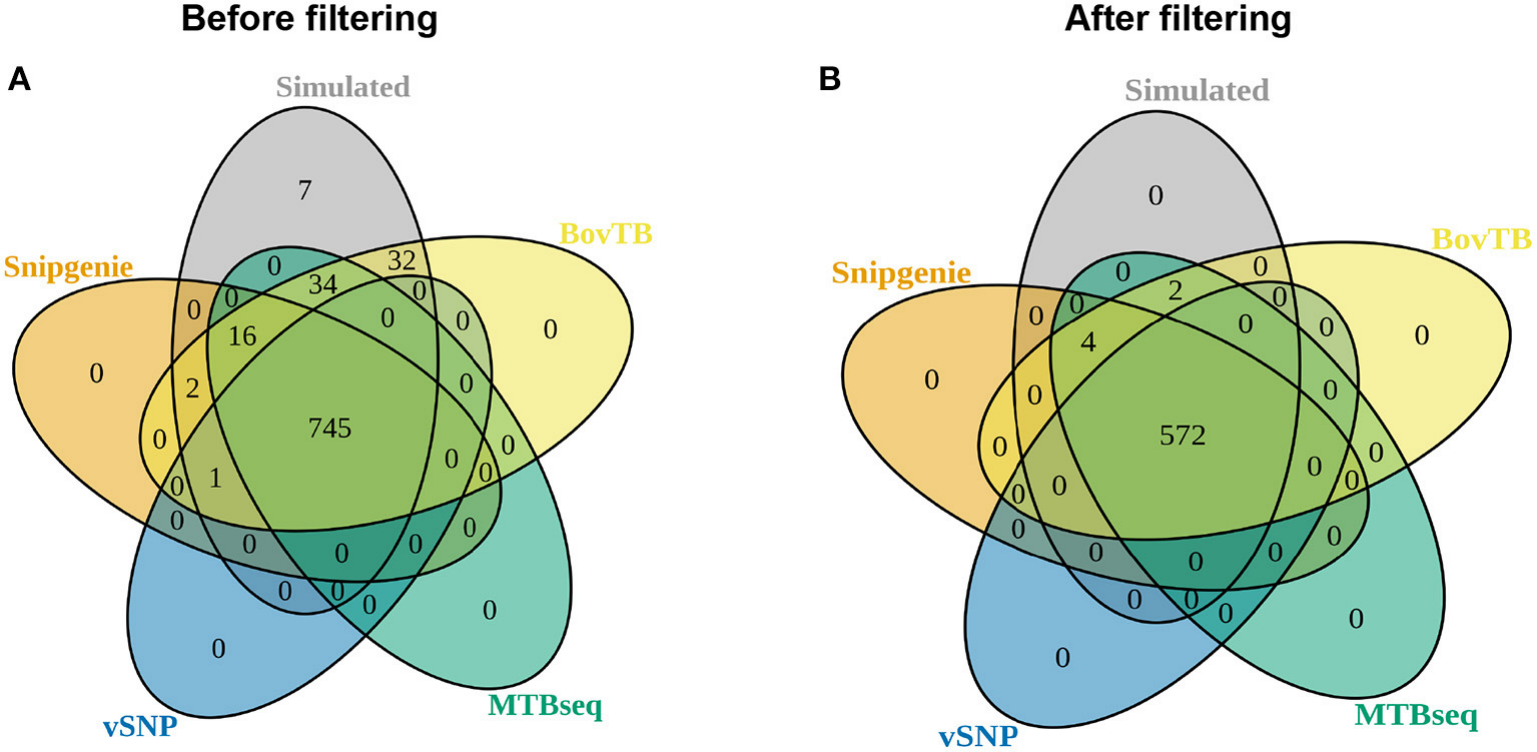
Venn diagram showing the agreement between the positions identified by the different pipelines and the simulated dataset **(A)** before and **(B)** after hard filters (filter F) were applied.

There was a high agreement between the SNPs identified by the different pipelines and those in the simulated genomes, with the majority of simulated SNP positions being appropriately detected (Figure 4A). When proximal SNPs and repetitive sequences were filtered (filter F), there was an increase in the agreement between pipelines (Figure 4B). An identical agreement was observed when repetitive sequences were filtered without the proximity filter (filter G) (data not shown). SNiPgenie, BovTB and MTBseq were able to identify all of the SNPs from the simulation, while vSNP was not able to detect 7 SNPs (Figure 4B).

### Tree Distance Comparison

**Figure 5:**
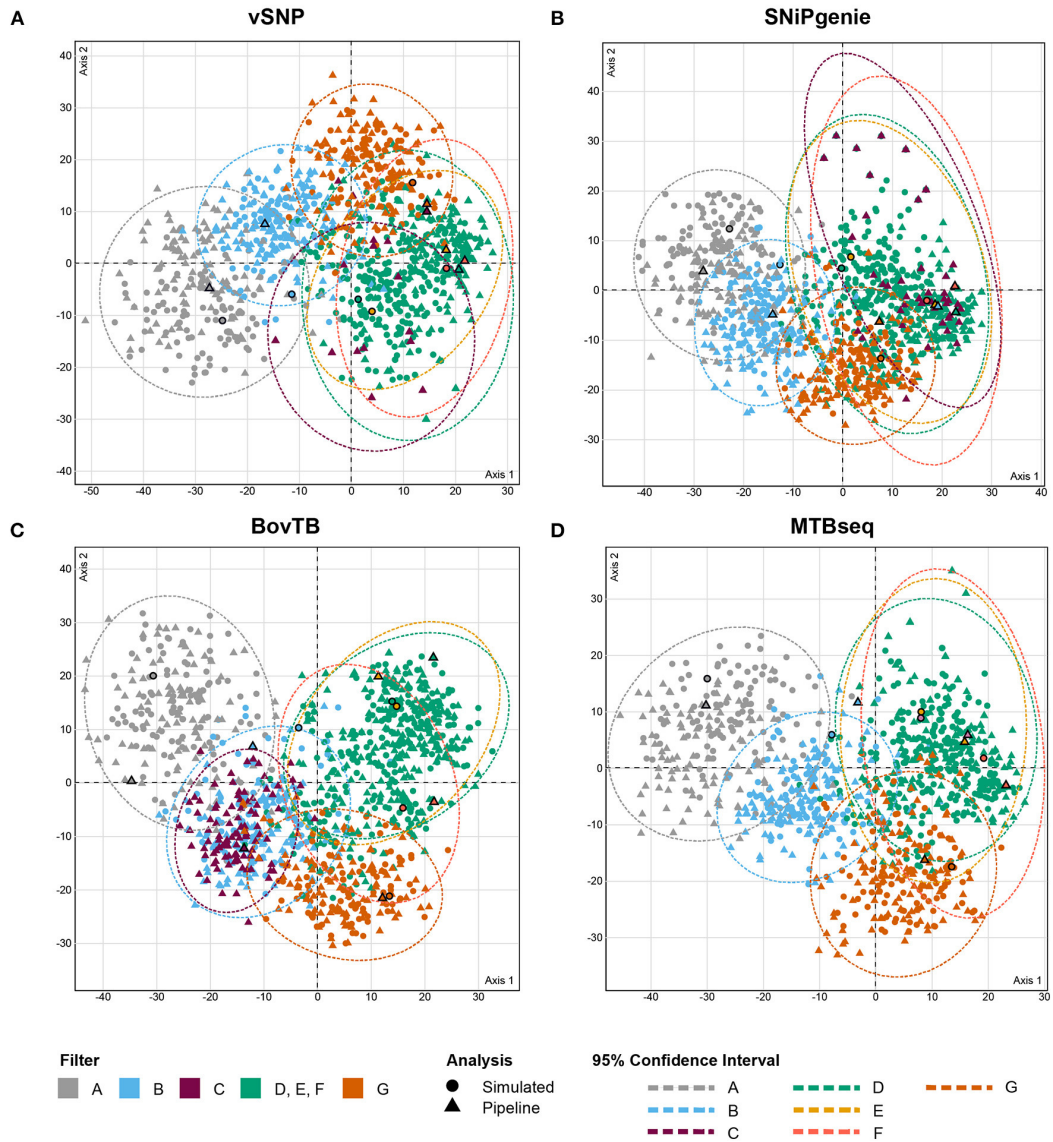
MCA analysis of the RF distances (first two dimensions) between maximum likelihood (ML) trees obtained from core SNP multi-FASTA alignments produced by **(A)** vSNP, **(B)** SNiPgenie, **(C)** BovTB, and **(D)** MTBseq. Shapes without outlines correspond to bootstrap replicates whereas bold shapes correspond to the best ML trees output by RAxML. Color shading corresponds to the hard filtering approach used.

The analysis of RF distances from best trees and bootstrap replicates revealed that trees output by the different pipelines clustered together with their simulated counterpart (Figure 5). In addition, cluster positioning was dependent on the type of hard filter used during the analysis. Trees obtained from the removal of problematic regions through filters D, E and F clustered together in one single group, whereas proximal filters (filter B) produced an intermediate clustering between unfiltered and filtered trees. The application of default filters (filter C) had an uneven effect in the different pipelines; BovTB trees did not separate considerably from proximally filtered trees (Figure 5C), whereas the trees produced by vSNP and SNiPgenie were closely related to other filtered trees.

### Pairwise Phylogenetic Comparisons

**Figure 6:**
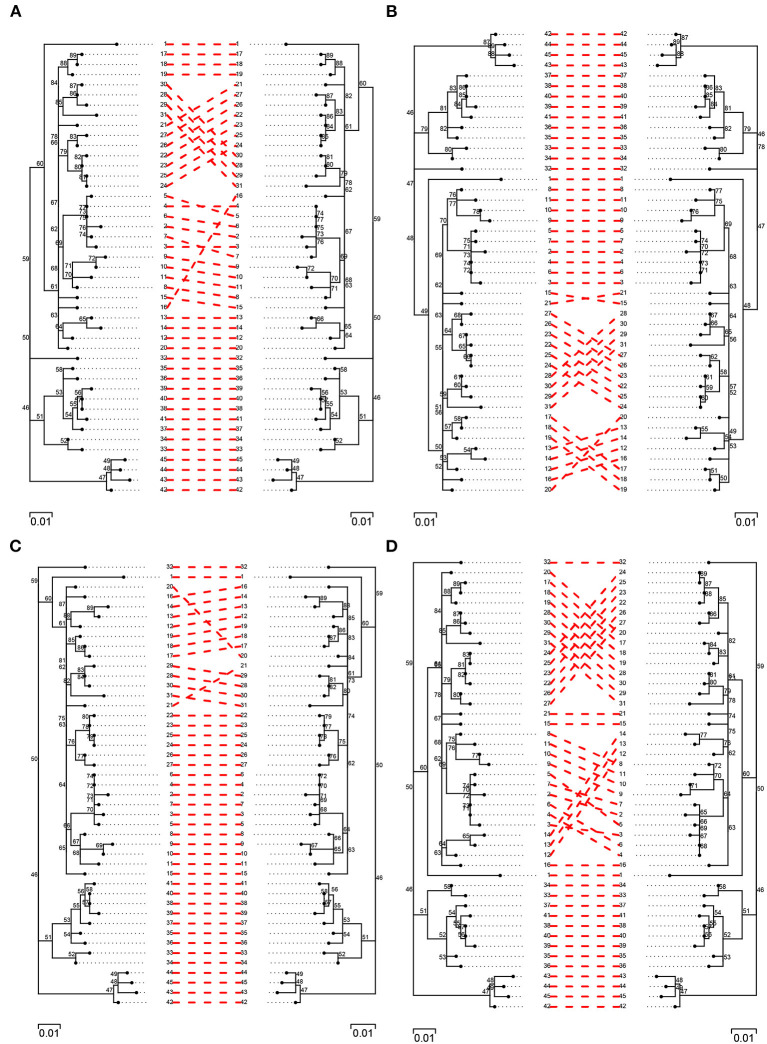
Pairwise comparison of filtered (filter F) simulated trees (left) and trees obtained from the evaluated pipelines (right): **(A)** vSNP, **(B)** SNiPgenie, **(C)** BovTB, and **(D)** MTBseq.

In order to further evaluate topological differences among trees, a pairwise comparison of best trees obtained from each of the pipelines was carried out against their simulated counterpart (Figure 6; Supplementary Figures 3, 4). In general, there was a high level of agreement among trees and pipelines, with agreement being highest among filtered trees and, especially, among those obtained from BovTB (Figure 6). Among default filtered trees, those obtained from SNiPgenie and BovTB presented a higher agreement with the simulation than vSNP (Supplementary Figure 4).

Three major groups of taxa could be identified in all trees and no inter-cluster exchange was observed between pipelines. Among unfiltered trees, several isolates presented a small change in their relative location within the tree in the different pipelines (e.g., isolates 9, 10 and 11 in vSNP, 17 and 18 in SNiPgenie, or 28 in MTBseq and BovTB), sharing their Most Recent Common Ancestor (MRCA) with a different group of isolates to the one observed in the simulation. The main differences among filtered trees were produced by small topological variations among highly related taxa (e.g., isolates 12, 13, and 14) and the appearance of polytomies further contributed to the topological differences observed with the simulated tree. When compared against filter F, filter G resolved a small number of polytomies (Supplementary Figure 5, blue squares). In all cases, the filtered trees were highly congruent with the topology represented in the original publication (Supplementary Figure 6).

## Discussion

The application of WGS technologies in the study of aTB has increased in the last decade around the world. Despite its great promise as a higher resolution alternative to traditional molecular techniques in phylogenetic and epidemiological studies, its implementation in the eradication of bTB is still in development.

The digital nature of the data produced by WGS platforms and tools facilitates the exchange of information between laboratories, fostering collaboration between countries and organizations tasked with aTB control. However, the plethora of tools, parameters, protocols and types of analyses available may introduce variations that hamper this process of communication. Standardized procedures and parameters are needed in order to reduce the effect of these variations.

Prior to any standardization taking place, there is a need to evaluate the currently available techniques. None of the variant calling pipelines designed up to date for aTB have been benchmarked in the scientific literature, leading to uncertainty regarding the best method to implement in laboratories that are considering incorporating WGS analyses into their workflows. The aim of this study was to carry out an evaluation of the performance of the currently available aTB variant calling pipelines and assess the degree of between-pipeline agreement in order to inform animal health authorities and laboratories.

The four pipelines evaluated in this study follow a similar procedure to other variant calling pipelines and employ bioinformatics tools widely used in the study of microorganisms. All pipelines use BWA as their sequence aligner (37), and the main procedural differences between them are related to the type of variant calling tools employed, and more importantly, the filtering process applied posteriorly.

The reduced sensitivity of vSNP, SNiPgenie, and MTBseq suggest that quality filters alone can negatively affect the performance of variant calling tools (21). The default minimum coverage settings of MTBseq, coupled with the minimum Allele Frequency of 75%, are important thresholds for the removal of possible strand bias but were not well suited for the simulated dataset at hand in which SNPs were present in the sample but strand bias was present. This highlights the importance of evaluating and adapting these parameters to the data being evaluated, as suggested by the developers (29). Discrepancies in performance between pipelines were related to a varying proportion of erroneous calls (FP and FN SNPs). In general, a low number of FP and FN SNPs were found in this study, in contrast with previous results in which a high number of erroneous calls were identified by different caller combinations used along with BWA for the analysis of *M. tuberculosis* sequences (21). This could be due to the different approach used in this study for simulated genome and read generation and pipeline-specific filters.

All of the FNs produced by vSNP, SNiPgenie, and MTBseq, and a small proportion of those produced by BovTB, were located within or near repeat-rich regions. A small number of FN positions were identified by all four pipelines and only one of these positions was due to a reduced coverage (<10). This suggests that differences in FN calls could be due to how sample-specific filters deal with low quality regions.

BovTB was the only pipeline to identify FP SNPs and an evaluation of these positions revealed that they were a result of being identified as mixed positions. These were, in addition, partially responsible for a small proportion of the FN SNPs and were effectively removed from the analysis during the consensus calling. As a result, these positions did not have any effect in posterior analyses.

Identified variants are usually translated into phylogenetic trees as a visual aid to assess the genetic relatedness between strains, which can help to identify epidemiologically related isolates suggestive of transmission. In this study, the phylogenetic trees obtained from the different pipelines clustered together with their respective simulated trees and a pairwise inspection revealed a high level of agreement between simulated and pipeline-specific trees, especially on those obtained from BovTB. The phylogenetic trees inferred from the unfiltered SNP alignments obtained from each pipeline were compared against the reference phylogeny in order to assess the effect of pipeline performance in phylogenetic inference. Small divergences were identified in vSNP, SNiPgenie, and MTBseq with respect to the simulation, which are probably a consequence of a reduced sensitivity due to the application of stringent quality filters alone.

Despite the abovementioned effects of quality filters in performance, these are rarely the only parameter taken into consideration when carrying out variant calling in MTBC species. Repeat-rich regions, such as PE/PPE family proteins, mobile genetic elements or direct repeats, are generally considered low confidence regions either due to a higher error rate or mapping issues (19, 38), which complicates the variant calling process and could give rise to FN and FP SNPs. Indeed, the majority of erroneous calls in our simulation were identified in repeat-rich sequences, especially in *pe/ppe* genes and the *pks12* gene.

In addition to the technical constraints that repetitive regions pose to sequencing procedures and mapping algorithms, these can also have a negative impact in phylogenetic inference due to the occurrence of homoplasies. These are genetic traits that can arise independently in separate lineages due to different causes, mainly as a result of convergent evolution but also as a consequence of sequencing and mapping errors. Homoplasic events can add varying grades of background noise in phylogenetic signals and, therefore, must be taken into consideration (39). Due to the limited genetic variation in *M. bovis* strains, this could be especially relevant in closely related isolates and could potentially alter the epidemiological conclusions drawn from outbreak investigations (40). Although homoplasies can be identified anywhere in the genome, they are more frequent in repeat-rich regions such as *pe/ppe* genes (41).

In our study, a small proportion of homoplasies were identified in the simulation, probably due to the reduced number of variant positions in our dataset in comparison with published literature (32). Unfiltered alignments obtained from the different pipelines contained a similar number of homoplasies, although vSNP, and SNiPgenie presented a slightly lower proportion, probably due to their more stringent quality filters. A large reduction in homoplasies was observed when proximity filters were applied and, although the subsequent filtering of repetitive sequences decreased homoplasies further, filtering of these sequences alone (filter G) led to an increase in homoplasies. This indicates that the proximity filter could be an important feature to decrease homoplasies outside the standard repetitive sequences.

Quality filters are, therefore, usually coupled with the removal of problematic regions, an approach nowadays considered a standard procedure in WGS analyses of MTBC species (19). There is, however, no current consensus as to which of these regions should be included in the hard filtering process. In our study, filtering out a progressive amount of regions increased the sensitivity of vSNP and SNiPgenie to levels similar to those observed for BovTB or MTBseq. This positive effect in performance was especially evident for *pe/ppe* genes and mobile genetic elements, and is probably a result of the increased weight of these sequences in the overall composition of the *M. bovis* genome (7–10%) (42). Interestingly, the use of proximal filters had a strong effect in the clustering of phylogenetic trees with a clear separation of these from unfiltered trees, which in turn could be due to the large reduction in homoplasies. Agreement between pipelines also improved with filtering, indicating a difference in the stringency in which the evaluated pipelines deal with problematic regions, and their dependency on posterior masking for removal of low confidence regions.

Hard filtering also had a positive effect in the agreement between phylogenetic trees, as was reflected by the reduced differences among the best ML trees. Although topological differences were identified, these were limited to a reduced number of isolates and polytomies and did not alter the relationship between isolates as seen in the original publication (22). These topological variations are probably related to the overall low bootstrap support values of the identified clusters (43), which in turn could be due to the limited genetic diversity observed in the original dataset in which our simulation is based on. Indeed, *M. bovis* isolates in the original publication presented a maximum of 35 SNPs with respect to each other and a median distance of 14 SNPs once all filters were applied (22). Such a reduced diversity reflects a common drawback encountered during *M. bovis* outbreak investigations, in which isolates from the same outbreak can accumulate a very small number of variants, hampering the definition of transmission events (26). Three different clusters were identified in our dataset in which genetic distances of *M. bovis* isolates were within 12 SNPs from each other; the maximum cut-off recommended for possible recent transmission of *M. tuberculosis* (44). In addition, polytomies can be resolved with increased isolate sampling, for example by including samples from wildlife or nearby breakdown events. However, this may not be a feasible option in many aTB outbreak investigations and, therefore, a removal of certain hard filters could be an interesting alternative to increase the amount of available informative SNPs. However, this alternative should be balanced to the risk of introducing possible biases or erroneous calls, such as FP SNPs. For example, although the removal of repetitive sequences without the proximity filter (filter G) increased the resolution of several polytomies, the increase in homoplasies could affect phylogenetic inference and needs to be considered.

There is little information as to how reliable low confidence regions are in phylogenetic inference, as their analysis has led to conflicting conclusions (45, 46). Nevertheless, there has been an increasing interest in the usefulness of filtering repeat-rich regions and recent data indicate that more than a half of the masked repetitive regions could be accurately identified using Illumina platforms (38). Even with the limitations of short-read sequencing platforms, the use of *de novo* assemblies or more refined masking filters may allow informative SNPs to be identified and retained (21, 38, 47). Furthermore, the introduction of long read sequencing could greatly improve the detection of variants within these regions of the genome (19). Improvements in the WGS analysis of problematic regions in MTBC species will surely benefit the field of aTB in the near future.

Pipeline choice may be based on other factors in addition to performance, and these have not been evaluated in this study. These include speed, use of disk space and memory, or ease of use, be it through the implementation of a GUI (SNiPgenie), limited command requirements (vSNP or BovTB) or by a straightforward data representation (vSNP and MTBseq) which could allow for more inexperienced users to access the bioinformatics analyses. In addition, the inclusion of additional analyses, such as antibiotic resistance profiling and cluster analysis (MTBseq), detection of INDELs and Regions of Difference (SNiPgenie), or lineage definition (vSNP, BovTB and MTBseq) could also be of interest for certain studies. However, in a similar manner to pipeline parameters, there is currently no standardized *M. bovis* lineage classification nor nomenclature based on WGS data. Although recent studies have suggested different lineages for *M. bovis* (48, 49), efforts toward this goal are still required. It is important to highlight that the results of our study are limited to simulated data and may not be representative of a real-life outbreak. The dataset used to generate our simulation does not correspond to an outbreak investigation but to a prevalence study. As a result, the capacity of each pipeline was approximated through their level of agreement with the simulation, rather than on their capacity to investigate true herd breakdown events. In addition, although this simulation partly mimics the negative impact of GC-rich sequences in genome coverage, it may be an underestimate in comparison to the actual sequencing of *M. bovis* isolates. Recent data highlight the existence of coverage blind spots in the *M. tuberculosis* reference genome which result from library preparation, sequencing as well as specific sequence attributes, such as homopolymers (50). Therefore, further work on a real-world dataset with a validated SNP profile and appropriate metadata is needed to evaluate these sources of bias.

Furthermore, the use of *M. bovis* AF2122/97 as a scaffold for the generation of simulated genomes meant that there were no sample-specific deletions, and therefore the capacity of these pipelines in calling SNPs near deletion events could not be evaluated. Furthermore, as is the case in human TB with *M. tuberculosis*, the choice of reference genome could also have an important effect in the WGS analysis of aTB due to differences in gene content between lineages, which could be masked by an inappropriate reference selection (19). This could be especially relevant when considering that traditional *M. bovis* lineages or clonal complexes are usually defined based on lineage-specific RDs, such as RDEu1 for the European 1 (Eu1) complex, or that certain genomic deletions may occur independently, such as the RD900 deletion. This study focused on the use of *M. bovis* AF2122/97, an Eu1 complex strain which is the default genome used by the evaluated aTB pipelines and the most extensively used *M. bovis* reference genome. However, the use of this reference genome in regions in which other clonal complexes are prevalent, such as the African 1 in western Africa, may lead to a loss of phylogenetic information. Therefore, other reference genomes may be better suited for different countries or regions and should be evaluated in the future.

Finally, it is important to note that, unlike other pipelines, manual and visual curation of SNPs is an important component of vSNP's design and functioning. As a result, a more detailed evaluation of this pipeline's results may have led to a reduced number of inconsistencies but would have added subjectivity to this comparison and was therefore avoided.

In conclusion, despite the above-mentioned limitations, the results of our comparison show that all evaluated pipelines perform well as long as similar hard filters are used, with minor differences amongst them with regard to performance and phylogenetic inference. This highlights the importance of standardizing and appropriately annotating filtering files when analyses are carried out between different laboratories or countries, and in particular in the context of aTB disease control.

## Data Availability Statement

The datasets presented in this study can be found in online repositories. The names of the repository/repositories and accession number(s) can be found below: https://doi.org/10.5281/zenodo.5179838, 5179838; https://github.com/Viloleal/bTB-pipeline-comparison-data-and-tools, None.

## Author Contributions

VL-L was involved in conceptualization, data curation, formal analysis, investigation, software, validation, and writing of the original draft. DF was involved in conceptualization, data curation, methodology, software, validation, and review and editing of the original draft. BR and LJ were involved in conceptualization, funding acquisition, and review and editing of the original draft. JÁ was involved in conceptualization and resources and review and editing of the original draft. SG was involved in conceptualization, funding acquisition, resources, validation, supervision, and review and editing of the original draft. All authors contributed to the article and approved the submitted version.

## Funding

The author VL-L was funded with a predoctoral grant from the Complutense University of Madrid and Banco Santander 2017–2018 (CT17/17—CT18/17). This research project was made possible thanks to an international fellowship grant from the Complutense University of Madrid and Banco Santander 2020 (EB25/20). DF and SG acknowledge funding from the Department of Agriculture Food and the Marine (DAFM) award 2019R404 (BTBGenIE). This project was partially funded by the EU-RL for Bovine Tuberculosis.

## Conflict of Interest

SNiPgenie was developed by DF and SG. The remaining authors declare that the research was conducted in the absence of any commercial or financial relationships that could be construed as a potential conflict of interest.

## Publisher's Note

All claims expressed in this article are solely those of the authors and do not necessarily represent those of their affiliated organizations, or those of the publisher, the editors and the reviewers. Any product that may be evaluated in this article, or claim that may be made by its manufacturer, is not guaranteed or endorsed by the publisher.
