# Supplementary material for: Performance and Agreement Between WGS Variant Calling Pipelines Used for Bovine Tuberculosis Control: Toward International Standardization
Source: Front Vet Sci. 2021 Dec 14;8:780018. doi: 10.3389/fvets.2021.780018 (PMC8712436; doi:10.3389/fvets.2021.780018)
Supplement: Supplementary file 1 [file Data_Sheet_1.pdf]

Supplementary Material

1 Supplementary Materials and methods

vSNP (USDA-APHIS)

The pipeline developed by the USDA-APHIS is written in python and run in separate steps using two different scripts (step 1 and step 2). In the first script, reads are aligned to a reference genome using BWA, followed by duplicate removal using Picard. Variant calling is then carried out using FreeBayes and an additional filtering step removes SNPs with a base quality < 20 from the obtained VCF files. Two types of VCF files are output during this initial step: standard VCFs and zero coverage VCFs that include those positions in the reference genome for which there is no read coverage. Additionally, unmapped reads can be analysed using ABySS and spoligotype is evaluated in silico and returned together with several statistics.

Zero coverage VCFs from all the analysed samples are input in the second script and quality thresholds are then used to determine whether a position will be reported in the output table, and whether this will be set as the reference call, an ambiguous IUPAC call or an alternate allele. A SNP position will be reported if at least one SNP in the dataset is called as an AC=2, QUAL value above 150 and MQ > 56. A SNP will be reported as IUPAC ambiguous (RYSWKM) if AC=1 and QUAL is above 150, N if QUAL is between 150 and 50, REF if QUAL is below 50, or as "-" if ALT is as None. Finally, all positions are aligned in a concatenated multi-FASTA file and in annotated excel tables for easy visualization.

SNPs that are shared among all of the evaluated samples (monomorphic SNPs) are removed from the output results and not considered during phylogenetic inference. Positions recorded in a filter excel file can be used to remove specific regions of the genome and/or cluster samples into specific genetic groups based on signature SNPs. Finally, step 2 runs RaxML to produce a Maximum-Likelihood tree from the multi-FASTA alignment file.

SNiPgenie (University College Dublin)

SNiPgenie is a pipeline written in python that includes an initial and optional read trimming step using cutadapt. Reads are then mapped against the reference genome using BWA mem and variants are called using BCFtools’ multiallelic caller. Variants are then filtered out using custom or default thresholds (QUAL>=40 && FORMAT/DP>=30 && DP4>=4 && MQ>30). Additionally, SNPs within a certain distance window (default = 10) from each other can be removed and specific genomic regions can be masked using a bed file. The remaining SNPs are then stored in VCF format and concatenated in a multifasta alignment file. When a variant is called in one sample, that position is considered as the reference call in those samples in which that variant is not present. An additional step can be activated to produce a phylogenetic tree using RaxML.

BovTB (Animal & Plant Health Agency)

BovTB is a pipeline written in nextflow developed by the Animal & Plant Health Agency in the UK for the analysis of M. bovis WGS data obtained from Illumina platforms. The pipeline initiates with a preprocessing step in which duplicate reads are removed using FastUniq and reads are trimmed with Trimmomatic. Reads are then mapped using BWA and variants are called using bcftools consensus caller. An additional step uses Kraken2 determine identity of reads when mapping statistics suggest a mixed culture or low quality sequence output.

During the run, known repeat regions in the M. bovis genome, as well as zero coverage regions, are masked from the analysis. Indels are normalised and SNPs with a depth < 5 and Allelic Fraction < 0.8 are removed. BovTB outputs a consensus genome applying the identified SNPs into the reference genome as well as a statistics file including lineage classification based on specific SNPs.

MTBseq

MTBseq is a pipeline written in Perl developed by the Research Centre Borstel – Leibniz Lung Centre. Its functioning is extensively described elsewhere (1) and will not be detailed here.

References

Kohl TA, Utpatel C, Schleusener V, De Filippo MR, Beckert P, Cirillo DM, et al. MTBseq: a comprehensive pipeline for whole genome sequence analysis of *Mycobacterium tuberculosis* complex isolates. PeerJ. 2018;6:e5895.

2 Supplementary Figures

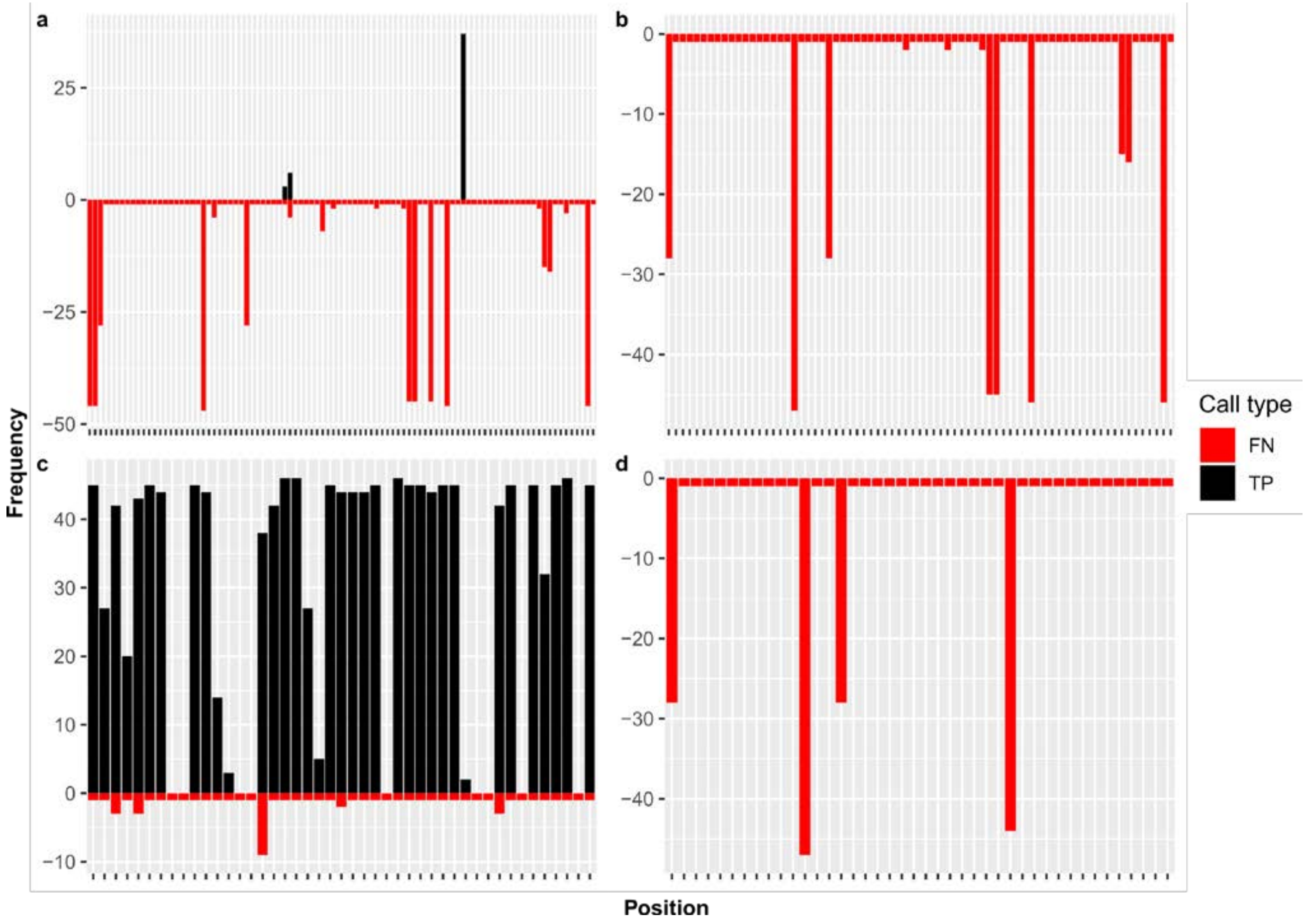

Supplementary figure 1. True SNP and FN SNP frequency across the genome positions identified in this study by the different pipelines: a) vSNP, b) SNiPgenie, c) BovTB and d) MTBseq.

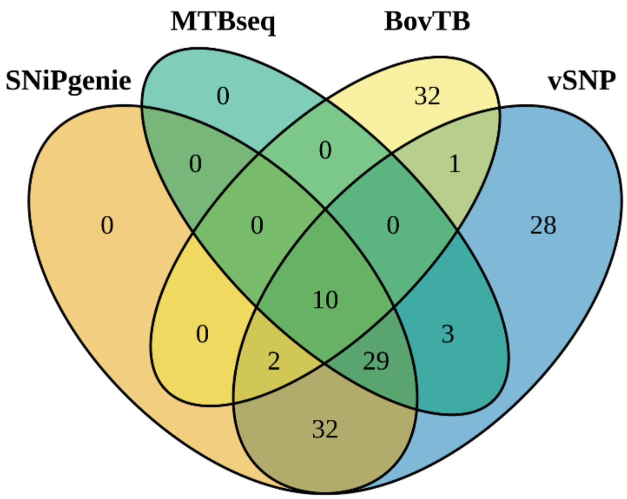

Supplementary figure 2. Venn diagram representation of unique and shared FN positions across the evaluated pipelines (unfiltered).

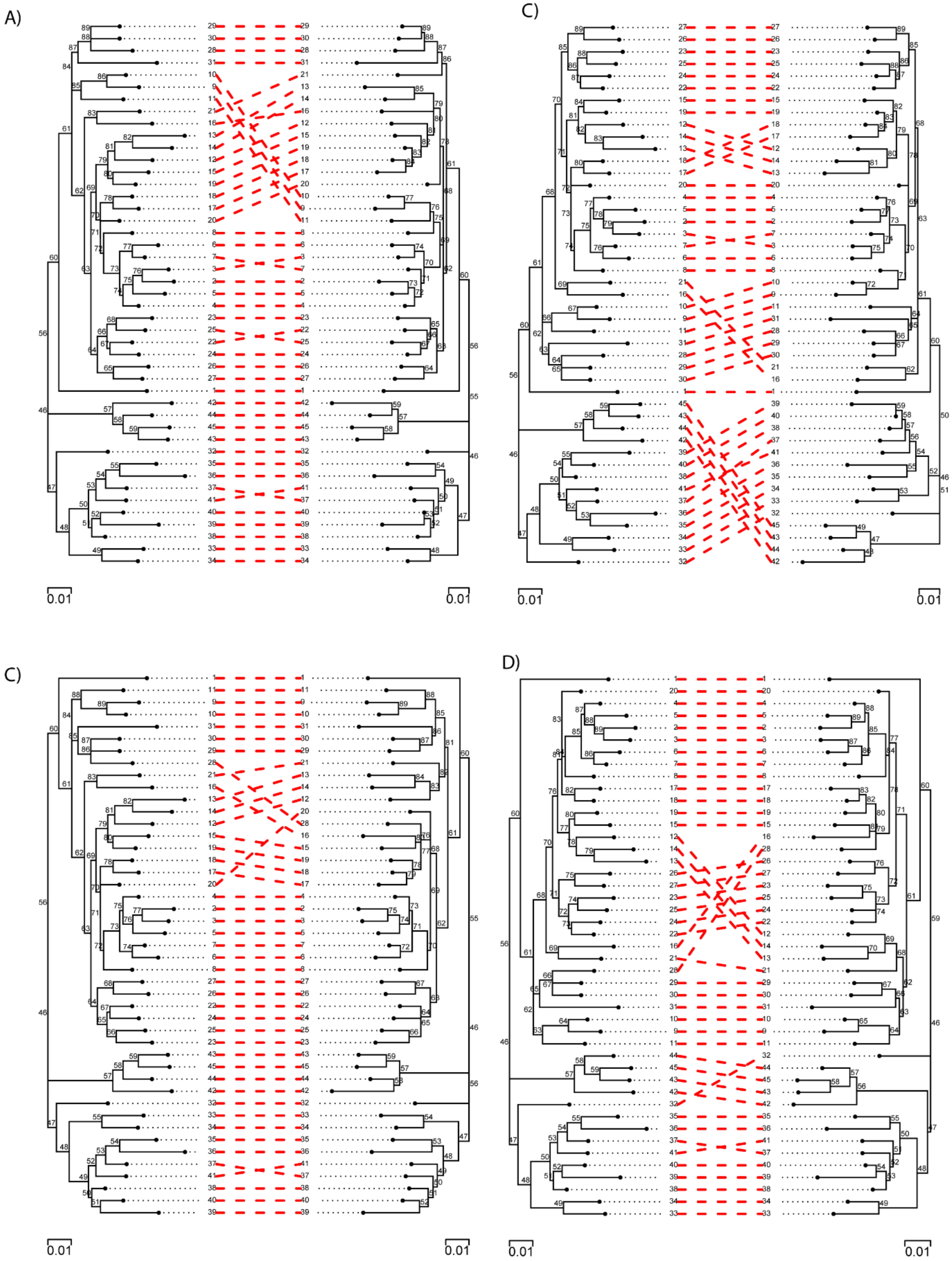

Supplementary figure 3. Pairwise topological comparison of unfiltered simulated best ML trees (left) and best ML trees obtained from the four evaluated pipelines (right): a) vSNP, b) SNiPgenie, c) BovTB and MTBseq.

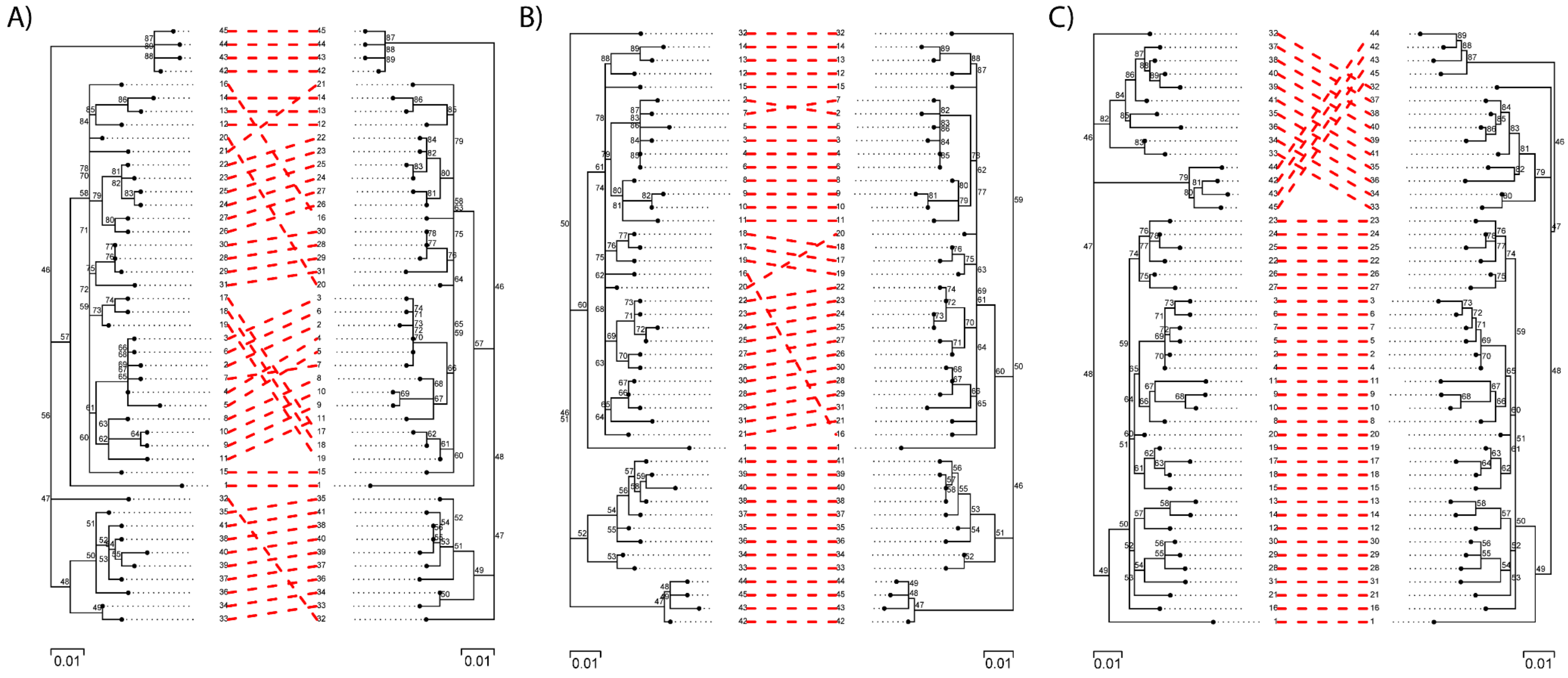

Supplementary figure 4. Pairwise topological comparison of simulated default filtered best ML trees from the simulated dataset (left) and default filtered best ML trees (right) obtained from a) vSNP, b) SNiPgenie and c) BovTB.

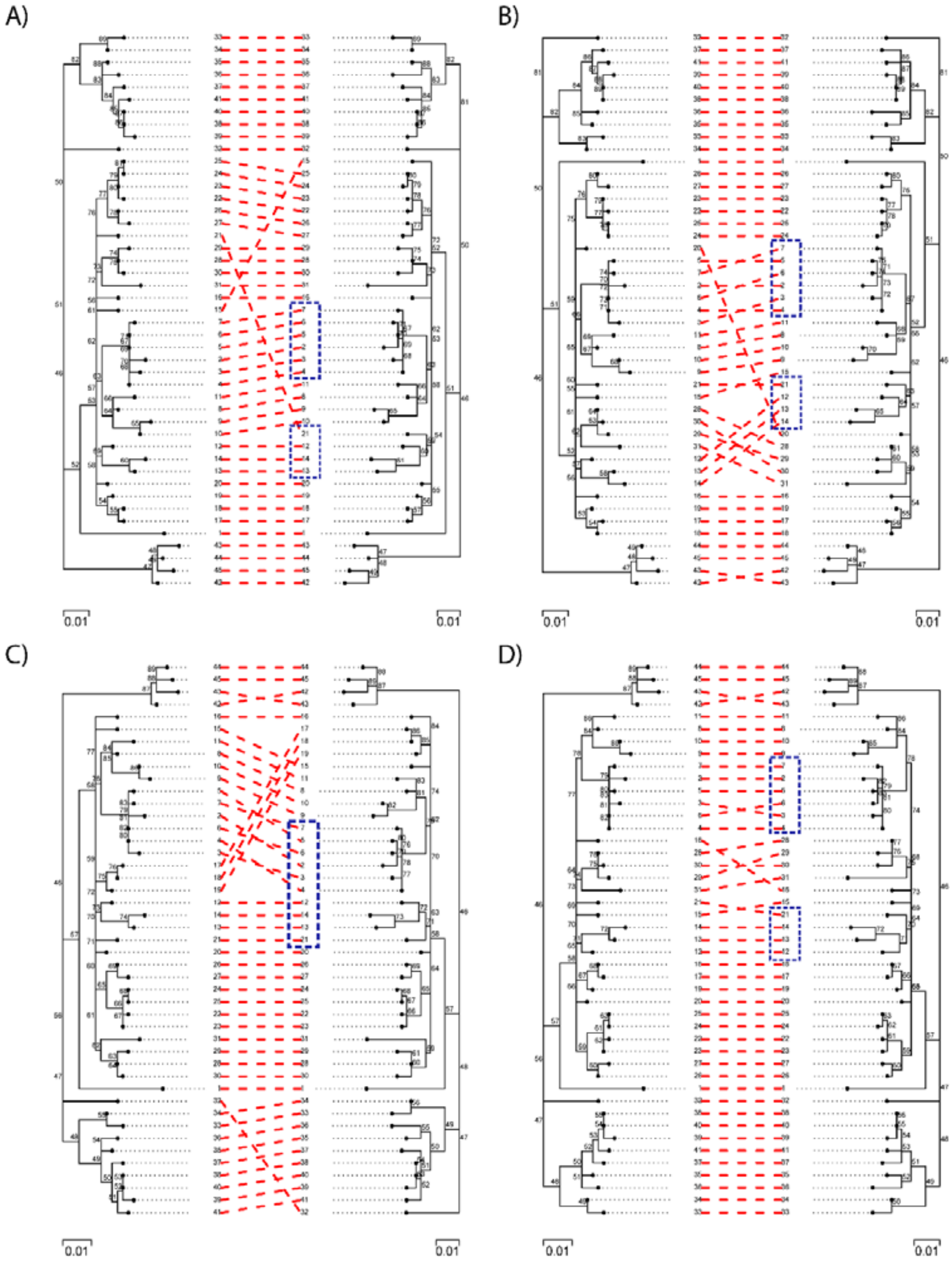

Supplementary figure 5. Pairwise topological comparison of filtered (filter F) best ML tress (left) and filtered (filter G) best ML trees (right) obtained from a) vSNP, b) SNiPgenie, c) BovTB and d) MTBseq.

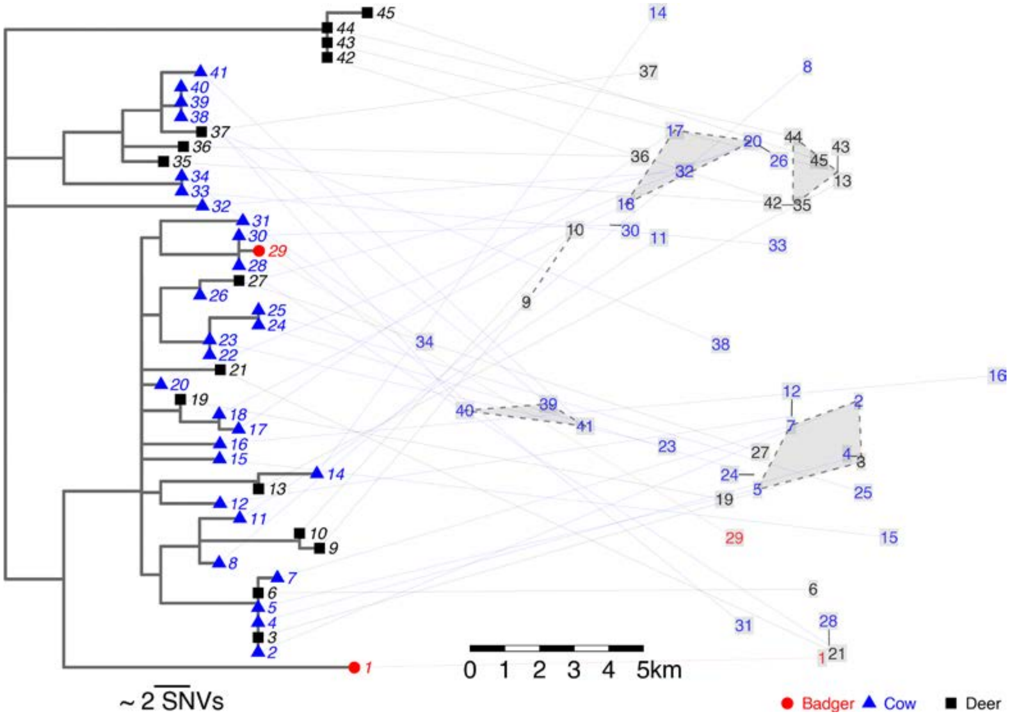

Supplementary figure 6. Original phylogeny in which the simulated dataset is based on. Tips are linked to their sampling locations via a solid line. Grey polygons represent clusters of isolates with three or less differences and with approximate sampling locations within 2.5 km. Extracted from (Crispell et al., 2020).

References

Crispell, J., Cassidy, S., Kenny, K., McGrath, G., Warde, S., Cameron, H., Rossi, G., MacWhite, T., White, P.C.L., Lycett, S., Kao, R.R., Moriarty, J., Gordon, S.V., 2020. *Mycobacterium bovis* genomics reveals transmission of infection between cattle and deer in Ireland. Microb Genom 6.
